# Sleepiness and attention in sleep-clinic patients and their associations with apnea severity and treatment

**DOI:** 10.1093/sleep/zsag094

**Published:** 2026-05-15

**Authors:** Allison Schwab, Brendan T Keenan, Nathan C Nowalk, Mathias Basner, Charles J Bae

**Affiliations:** Division of Sleep Medicine/Department of Medicine, University of Pennsylvania Perelman School of Medicine, Philadelphia, PA, United States; Division of Sleep Medicine/Department of Medicine, University of Pennsylvania Perelman School of Medicine, Philadelphia, PA, United States; Division of Sleep Medicine/Department of Medicine, University of Pennsylvania Perelman School of Medicine, Philadelphia, PA, United States; Unit for Experimental Psychiatry, Division of Sleep and Chronobiology, University of Pennsylvania Perelman School of Medicine, Philadelphia, PA, United States; Division of Sleep Medicine/Department of Medicine, University of Pennsylvania Perelman School of Medicine, Philadelphia, PA, United States

**Keywords:** PVT, psychomotor vigilance test, OSA, sleep apnea, sleepiness, alertness, Epworth Sleepiness Scale, CPAP

## Abstract

**Study Objectives:**

Excessive daytime sleepiness (EDS) is common in patients with sleep disorders, particularly obstructive sleep apnea (OSA). The Epworth Sleepiness Scale (ESS) and the Psychomotor Vigilance Test (PVT) are often used to measure sleepiness and attention/vigilance, respectively. However, whether these measures are correlated and their relationship to OSA severity/treatment remains understudied. This study examined these associations in a sleep center population.

**Methods:**

We performed a prospective study of 165 participants (119 [72.1%] with diagnosed OSA) presenting to a sleep disorders clinic and completing ESS and PVT. Associations among ESS, PVT, OSA severity, and positive airway pressure (PAP) usage and efficacy were examined using Pearson’s correlations, unadjusted and controlling for age, sex, and body mass index.

**Results:**

Results showed no significant correlations between ESS and PVT measures of attention/vigilance. While higher ESS scores correlated with more severe apnea–hypopnea indices in patients with OSA, no association was found with OSA severity and PVT measures. Among participants using PAP, greater hours/night of usage was associated with lower ESS scores, but not with better PVT performance.

**Conclusions:**

We found no association between sleepiness on the ESS and attention/vigilance on PVT among a sleep center population. ESS scores tracked more closely to OSA severity/treatment than PVT measures. The findings suggest that the sleepiness measured by the ESS and measures of sustained attention under monotonous conditions from the PVT capture distinct symptoms. While the ESS is commonly used, further research is needed to determine if the PVT should also be incorporated into clinical practice.

## Graphical Abstract

**Graphical Abstract:**
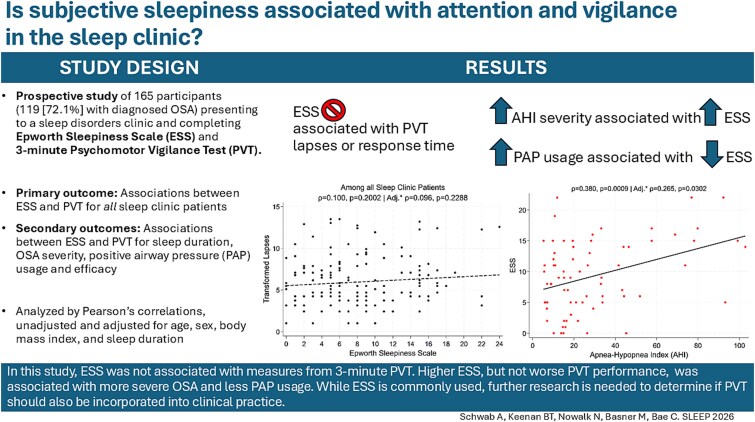

Statement of Significance Excessive daytime sleepiness (EDS) is a common symptom among individuals presenting to a sleep clinic, but the relationship between subjective sleepiness (Epworth Sleepiness Scale) and objectively measured attention/vigilance (Psychomotor Vigilance Test, PVT), as well as how each measure relates to obstructive sleep apnea (OSA) severity and treatment, is not well understood. This study found no association between the ESS and measures from a 3-minute PVT, suggesting that these assessments are evaluating different symptoms experienced by participants. Higher ESS score, but not worse PVT performance, was related to more severe OSA and less usage of positive airway pressure, indicating that the ESS tracks more closely than the PVT to OSA severity and treatment.

## Introduction

Among patients with sleep disorders, excessive daytime sleepiness (EDS) is a frequently reported symptom that can lead to a decrease in cognitive performance, mood, and overall wellbeing [1]. EDS is commonly measured by the Epworth Sleepiness Scale (ESS), a self-reported questionnaire assessing the likelihood of dozing off or falling asleep across common activities [2, 3]. While the ESS measures daytime sleepiness, it has also been used to screen patients for obstructive sleep apnea (OSA), as EDS is a very common symptom of OSA [4]. In patients with OSA, greater sleepiness on the ESS is associated with higher blood pressure and cardiovascular disease risk [5–11].

Although the ESS is the most commonly used metric for characterizing EDS in patients with OSA, it only has moderate utility to screen for OSA [12]. Data are also mixed on how well the ESS associates with OSA severity based on metrics such as the Apnea–Hypopnea Index (AHI) [13, 14]. This suggests additional measures of EDS may help to quantify a patient’s symptoms more accurately. For example, recent work has suggested that combining responses to a single question on feeling sleepy during the day with the ESS can identify patients with worse quality of life and more OSA-related symptomatology [15]. There is also a growing literature on OSA symptom–based subtypes, one of which (termed *excessively sleepy*) is defined by the presence of multiple symptoms of excessive sleepiness and may account for the elevated cardiovascular risk related to OSA [6–8, 10, 16–19]. Notably, the ESS alone is insufficient to identify patients with this subtype [19, 20].

Measuring EDS objectively may provide a better representation of the impact of daytime sleepiness among patients with OSA. Toward this end, the psychomotor vigilance test (PVT) provides objective measures of deficits in attention and vigilance [21, 22]. The PVT is a reaction time test that has been used to measure deficits of sleep loss, such as optimal neurobehavioral functioning in astronauts [23], screening for impaired vigilance in commercial drivers and emergency responders [24], and poor sleep quality affecting medical attentiveness in nurses [25, 26]. The PVT is highly sensitive to small changes in attention, which can occur from sleep deprivation and other sleep disorders [27]. Additionally, the PVT has no learning curve and its reliability and validity have been proven by previous studies [28, 29].

Prior studies have found that the PVT and ESS are correlated, with higher ESS scores associated with a greater number of lapses in untreated participants with OSA [30–32]. However, these findings have not been replicated in a clinical sleep setting. To better understand the measurement of “sleepiness,” this study examines the relationship between the ESS and PVT measures in a sample of patients from a clinical sleep center. Moreover, among those with diagnosed OSA, we examined how each measure associates with disease severity and Continuous Positive Airway Pressure (CPAP) usage (hours/night of use) and efficacy (mask leak and residual AHI) among those using treatment. While previous studies have shown that adherence to CPAP can improve patient-reported sleepiness, how sleepiness relates to measures like residual AHI and mask leak is a less well-studied but clinically important question [33].

We hypothesized that the ESS and PVT measures would be correlated among patients presenting to the sleep clinic. Among those with OSA, we expected the PVT (since it is an objective measure) would be more strongly correlated with AHI severity and the usage and efficacy of CPAP than the subjective ESS. If this were the case, it would support incorporating a PVT as a standard metric in participants being evaluated and/or treated for OSA. Regardless of the relationships with ESS or OSA severity/treatment, incorporating the PVT into routine clinical practice may provide a new outcome measure for all sleep disorders (e.g. narcolepsy, insomnia, restless leg syndrome, periodic limb movements).

## Materials and Methods

### Subjects

This investigation is based on data gathered prospectively from a consecutively enrolled clinical sample of patients evaluated in the Sleep Center at the University of Pennsylvania over a 3-month period (June 2022 to August 2022). The sample consisted of individuals who completed a PVT and ESS during their clinic visit. Inclusion criteria were intentionally broad to reflect typical patients presenting to the sleep clinic. The exclusion criteria were missing information on either PVT or ESS (e.g. due to the physician not obtaining ESS or the patient not having time to complete PVT during their visit) or an inability to complete these tasks (e.g. cognitive impairment or language barrier). Additional data were extracted from the electronic health record (EHR). This project was reviewed and determined to be Quality Improvement by the University of Pennsylvania’s Institutional Review Board.

### Epworth Sleepiness Scale

Patients completed the Epworth Sleepiness Scale (ESS) at the beginning of their clinical visit. The ESS includes eight questions asking the patient to rate their tendency to doze or fall asleep (on a scale of 0–3) across eight common and varied situations [2]. The total score of the ESS is calculated by adding up patient responses to each question and can range between 0 and 24, with a score > 10 commonly used to indicate excessive daytime sleepiness.

### Psychomotor Vigilance Test

At the conclusion of the clinical visit, patients completed the 3-minute Psychomotor Vigilance Test (PVT) [34] on an HP Mobile Workstation ZBook 15 G3 15.6-inch FHD Laptop. The PVT is an attention/reaction time test in which participants are tasked with pressing a button as soon as a randomly timed visual stimulus appears. The PVT measures the speed and accuracy with which participants respond to the stimuli, providing an objective measure of alertness. The PVT has been studied as 10-, 5-, and 3-minute versions with our study electing to use the latter for the time constraints of the clinical encounter. Primary measures from PVT include the number of lapses (defined as a response time [RT] >355 ms), which is transformed as square root of lapses plus the square root of lapses +1 for analysis, and the mean reciprocal response time (RRT) [27]. The sampling rate (the time interval before the stimulus is presented) is randomized between 2000 and 5000 ms.

### Sleep Duration

Sleep duration was derived from an electronic health record (EHR) chart review. Patients self-reported habitual sleep schedules as part of routine clinical care, from which sleep duration was calculated. A total of 134 (81.2%) of 165 participants had sleep duration information available from encounters on the same day as PVT and ESS assessments.

### Sleep Studies

**Figure 1:**
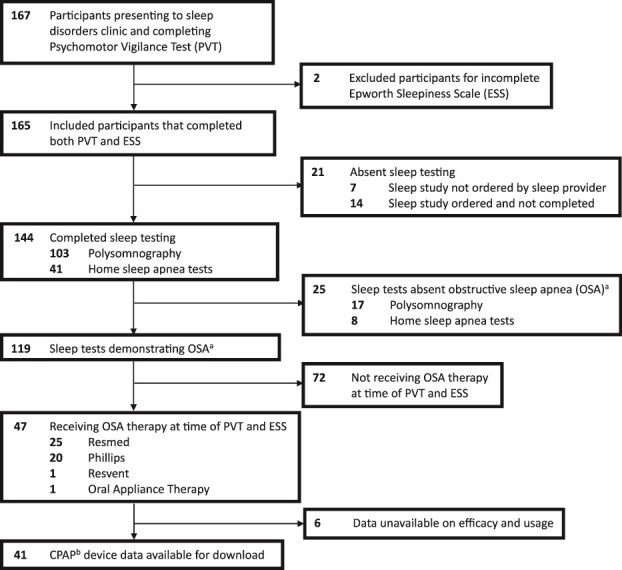
Patient flow diagram.

Of the 165 eligible patients, 144 had sleep studies, including 103 (71.5%) in-laboratory polysomnography (PSG; Philips G3) studies and 41 (28.5%) home sleep apnea tests (HSATs) (Figure 1). Twenty-one patients did not have complete sleep studies: 7 did not require sleep study, and 14 had a sleep study ordered but never completed. During the HSAT (Philips NightOne or Clevemed) nasal airflow, chest and abdominal movements, pulse, oxygen saturation, body position, and body movement were all measured. A total of 136 of 144 sleep studies (94.4%) were scored using the following criteria: apneas were defined as an absence of airflow for >10 seconds and hypopneas as a 30 per cent reduction in airflow for >10 seconds associated with a ≥4% decrement in oxygen saturation. The remaining eight sleep studies (OSA: 7; Absent OSA: 1) were performed by outside sleep laboratories using unclear scoring criteria and were not available for review of the raw data. For the purposes of our primary analysis, and consistent with clinical practice, the respiratory event index (REI) derived from HSAT was considered the participants’ AHI. Participants were classified as having OSA if sleep testing demonstrated an AHI >5 events/hour with a majority of obstructive respiratory events (<50% central respiratory events).

### Continuous Positive Airway Pressure

Of the 119 patients with OSA, 47 (39.8%) were known to be using an OSA therapy before their clinical visit: 46 using CPAP (25 on ResMed devices, 20 on Philips devices, and 1 on a Resvent device) and 1 using an oral appliance. Data on CPAP usage and/or efficacy were available on 41 (89.1%) of the 46 patients using CPAP (from Philips and ResMed databases), including measures of the average usage (hours/night) in the 1 month, 1 week, and 1 day prior to the visit, as well as the residual AHI (events/hour) and device-specific leak measures (time in large leak from Philips devices and 95th percentile leak from ResMed devices) over the 1 month before the visit. Mask leak data were included to provide context in interpreting the residual AHI, as excessive leak can impair event detection and lead to underestimation of residual AHI [35, 36].

### Statistical Methods

Continuous variables are summarized using means and standard deviations (SDs) and categorical variables using frequencies and percentages. Where presented, comparisons of demographic variables between groups were performed using *t*-tests (continuous) and chi-square or Fisher’s exact tests (categorical). To examine the associations between ESS and PVT measures, we utilized Pearson’s linear correlations and partial correlations adjusted for age, sex, and body mass index (BMI). Associations were examined among all patients and separately for the subset of patients with diagnosed OSA. To assess the impact of treated OSA on the primary findings, these analyses were repeated after excluding patients receiving OSA therapy. Similar analyses were used to examine the associations between OSA severity and sleepiness/attention measures among patients with OSA with available AHI not using therapy, as well as to evaluate associations between CPAP usage (hours/night), residual AHI, or leak metrics and sleepiness/attention measures among patients with OSA in whom CPAP data were available. In sensitivity analyses examining correlations between OSA severity and sleepiness measures, correlations were repeated stratified by sleep study type (HSAT or PSG). Lastly, associations between self-reported sleep duration and ESS, PVT, OSA severity, CPAP usage, and efficacy were examined, after which sleep duration was added as a covariate to the prior models evaluating the relationship of OSA and CPAP metrics with ESS or PVT. As defined by Cohen [37], correlation coefficients of 0.1, 0.3, and 0.5 are interpreted as small, medium, and large effect sizes. A Hochberg step-up procedure was utilized to determine statistical significances while maintaining type I error at 5% within the context of multiple comparisons performed in each of the above analyses [38, 39].

## Results

### Sample Characteristics

A total of 165 participants were included in this study (Table 1; Figure 1). Participants were middle-aged (55.2 ± 16.9 years), obese (BMI of 34.5 ± 9.9 kg/m^2^), a majority were males (59.4%), and there was a similar proportion of black (44.6%) and white (42%) participants. Of the 165 participants, 119 (72.1%) had diagnosed OSA. Five participants had a central apnea index (CAI) >5 events/hour; however, no participant met the criteria for central sleep apnea, defined as >50% respiratory events central in etiology.

**Table 1:** Descriptive characteristics of the analysis sample

Measure	All
*N*	165
Age (years)	55.2 ± 16.9
Male	59.4%
BMI (kg/m^2^)	34.5 ± 9.9
Weight (pounds)	222.9 ± 66.4
Height (inches)	67.5 ± 4.0
Race	
White	42.0%
Black	44.6%
Asian	5.1%
Other	8.3%
Sleep diagnosis	
OSA	72.1%
Snoring	6.1%
Insomnia	7.9%
Idiopathic hypersomnia	1.2%
Parasomnias including RBD	2.4%
Narcolepsy	0.6%
Other^*^	3.0%
Untested	6.7%
ESS	8.07 ± 5.39
PVT lapses	10.5 ± 10.6
PVT transformed lapses	5.95 ± 2.90
PVT mean RRT	3.40 ± 0.69
Sleep duration (hours)	6.99 ± 1.65

^*^Other diagnoses (circadian rhythm disorders, poor sleep hygiene, myopathy); Abbreviations: BMI = Body Mass Index; RBD = REM behavior disorder; PVT = Psychomotor Vigilance Test; ESS = Epworth Sleepiness Scale; RRT = Reciprocal Response Time

Similarly, characteristics of patients with diagnosed OSA (*n* = 119) were stratified by whether or not the participant was using an OSA therapy prior to their clinical visit (Table 2). On average, the 47 (39.8%) patients on therapy were about 8 years older (62.4 ± 15.6 years) than those not (54.6 ± 14.9 years; *p* = .008), but other demographic characteristics were generally similar. Those with OSA therapy had some evidence of less severe hypoxemia but no significant difference in AHI. Out of the 46 patients using CPAP, objective usage and/or efficacy data were available for 41 (89.1%). These patients used CPAP for an average of 5.90 ± 2.18 hours/night over the last 30 days. The median (IQR) residual AHI was 3.1 (1.3, 5.2) events/hour, the time in large leak for those on Philips devices was 13.9 (0.6, 93.6) minutes, and the 95th percentile leak for those on ResMed devices was 20.8 (8.5, 41.2) L/min.

**Table 2:** Descriptive characteristics of participants with OSA using and not using CPAP at the time of the clinical visit

Measure	OSA	No treatment	**Treatment** ^*^	*P*
*N*	119 (100%)	72 (60.2%)	47 (39.8%)	–
Age (years)	57.7 ± 15.6	54.6 ± 14.9	62.4 ± 15.6	.008
Male	66.4%	65.3%	68.1%	.751
BMI (kg/m^2^)	35.7 ± 9.9	36.0 ± 10.7	35.1 ± 8.7	.630
Weight (pounds)	232.7 ± 67.9	235.7 ± 71.8	228.2 ± 61.9	.551
Height (inches)	67.8 ± 4.0	68.0 ± 4.2	67.4 ± 3.8	.468
Race				.347
White	38.3%	31.9%	47.8%	
Black	46.1%	52.2%	37.0%	
Asian	6.1%	5.8%	6.5%	
Other	9.6%	10.1%	8.7%	
AHI (events/hour)	32.9 ± 27.7	29.4 ± 25.0	38.2 ± 31.0	.107
SpO_2_ nadir (%)	78.9 ± 8.3	77.5 ± 8.5	81.0 ± 7.7	.029
Minutes with SpO_2_ < 90%	38.7 ± 68.0	52.2 ± 80.2	16.8 ± 30.7	.001
ESS	8.52 ± 5.48	9.26 ± 5.80	7.38 ± 4.80	.057
PVT lapses	10.2 ± 10.9	10.5 ± 11.4	9.9 ± 10.2	.771
PVT transformed lapses	5.83 ± 2.98	5.90 ± 3.01	5.73 ± 2.96	.756
PVT mean RRT	3.41 ± 0.75	3.37 ± 0.82	3.47 ± 0.64	.449
Sleep duration (hours)	6.92 ± 1.62	6.78 ± 1.83	7.17 ± 1.13	.211

Abbreviations: BMI = Body Mass Index; AHI = Apnea Hypopnea Index; ODI = Oxygen Desaturation Index; PVT = Psychomotor Vigilance Test; ESS = Epworth Sleepiness Scale; RRT = Reciprocal Response Time.

^*^Includes *n* = 46 patients known to be on CPAP (data on average usages available in *n* = 41) and *n* = 1 patient on an oral appliance at the time of PVT.

### Association between ESS and PVT

**Figure 2:**
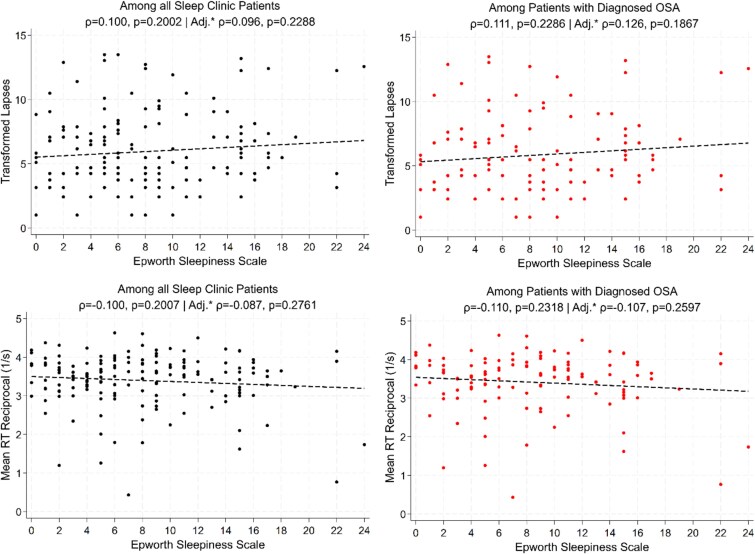
Associations among sleepiness and attention/vigilance measures and OSA severity*.* The associations between the Epworth Sleepiness Scale (ESS) and Psychomotor Vigilance Test (PVT) measures of (A) transformed lapses and (B) mean reciprocal response time (RRT) are illustrated in all participants and separately in those with diagnosed OSA. The data showed no meaningful correlations between subjective sleepiness and either PVT measurement for OSA and Non-OSA participants (see also Table 3). ^*^Adjusted for age, sex, and BMI.

Participants had an average ESS of 8.07 ± 5.39 and averaged 10.5 ± 10.6 lapses (5.95 ± 2.90 transformed lapses) and a mean RRT of 3.40 ± 0.69 seconds^−1^ on the PVT (Table 1). Among all participants, there were no significant correlations between ESS and either transformed lapses (adjusted rho = 0.10, *p* = .229) or mean RRT (adjusted rho = −0.09, *p* = .276); results and interpretations were similar when restricted to patients with diagnosed OSA (Table 3; Figure 2) and after excluding patients with treated OSA (Table S1).

**Table 3:** Correlations between ESS and PVT measurements

PVT measure	ESS of all participants				ESS of diagnosed OSA			
	*Unadjusted*		***Adjusted*** ^*^		*Unadjusted*		***Adjusted*** ^*^	
	*rho*	*P*	*rho*	*P*	*rho*	*P*	*rho*	*P*
Transformed lapses	0.10	.200	0.10	.229	0.11	.229	0.13	.187
Mean RRT	−0.10	.201	−0.09	.276	−0.11	.232	−0.11	.260

Abbreviations: PVT = Psychomotor Vigilance Test; ESS = Epworth Sleepiness Scale; RRT = Reciprocal Response Time.

^*^Partial correlation adjusted for age, sex, and BMI.

### Associations with OSA Severity

**Figure 3:**
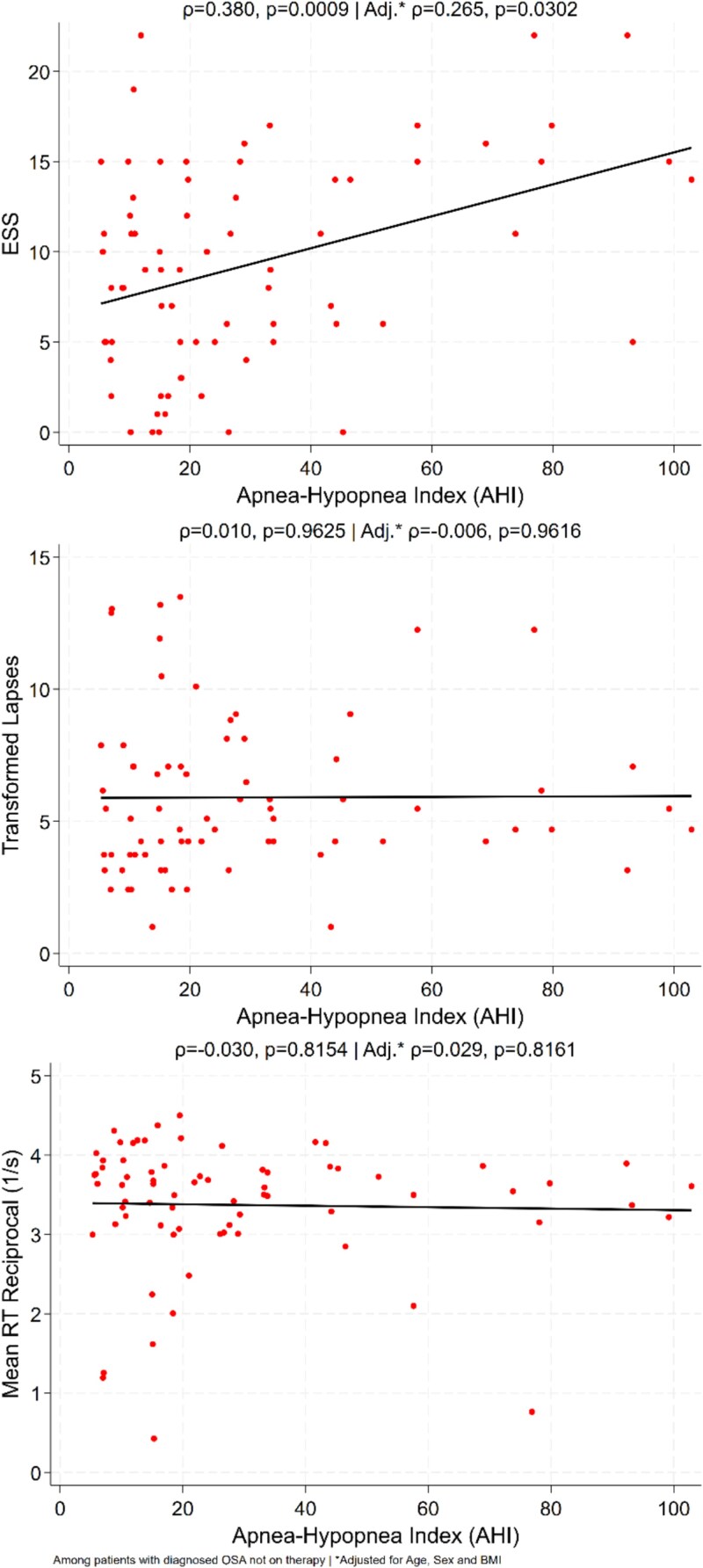
Associations between Apnea–Hypopnea Index (AHI) and sleepiness or attention/vigilance among participants with OSA not using therapy. We found significant correlations between AHI and sleepiness based on ESS, but no relationship between AHI and measures of attention/vigilance from PVT (see also Table 4). ^*^Adjusted for age, sex, and BMI.

Associations between measures of OSA severity and sleepiness were also evaluated among patients with OSA not on therapy (Table 4**;**  Figure 3). More severe OSA was generally associated with higher ESS scores based on the AHI (adjusted rho = 0.27, *p* = .030) or minutes SpO_2_ < 90% (adjusted rho = 0.31, *p* = .015); the association with AHI was not statistically significant after Hochberg correction but represents a moderate correlation. None of the OSA severity measures were significantly correlated with PVT-based measures of attention/vigilance. Results were generally consistent when stratified by type of sleep study, with slightly stronger correlation coefficients with ESS for severity metrics from HSAT and higher unadjusted correlations with PVT on PSG; results were not statistically significant in the reduced sample sizes (Table S2).

**Table 4:** Correlations between OSA Severity and both ESS and PVT measurements

Measure	AHI				SpO_2_ nadir				Minutes SpO_2_ < 90%^†^			
	*Unadjusted*		Adjusted^*^		*Unadjusted*		Adjusted^*^		*Unadjusted*		Adjusted^*^	
	*rho*	*P*	*rho*	*P*	*rho*	*P*	*rho*	*P*	*rho*	*P*	*rho*	*P*
ESS	**0.38**	**.001**	0.27	.030	**−0.32**	**.008**	−0.18	.170	**0.36**	**.003**	**0.31**	**.015**
Transformed lapses	0.01	.963	−0.01	.962	−0.18	.132	−0.18	.157	0.13	.277	0.12	.358
Mean RRT	−0.03	.815	0.03	.816	0.16	.180	0.12	.370	−0.10	.429	−0.04	.737

Statistically significant associations after Hochberg correction for three sleepiness measures shown in **bold**. Abbreviations: PVT = Psychomotor Vigilance Test; ESS = Epworth Sleepiness Scale; RRT = Reciprocal Response Time.

^*^Partial correlation adjusted for age, sex, and BMI;

^†^Natural log-transformed for analyses.

### Effects of CPAP Usage on ESS Scores and PVT Performance

**Figure 4:**
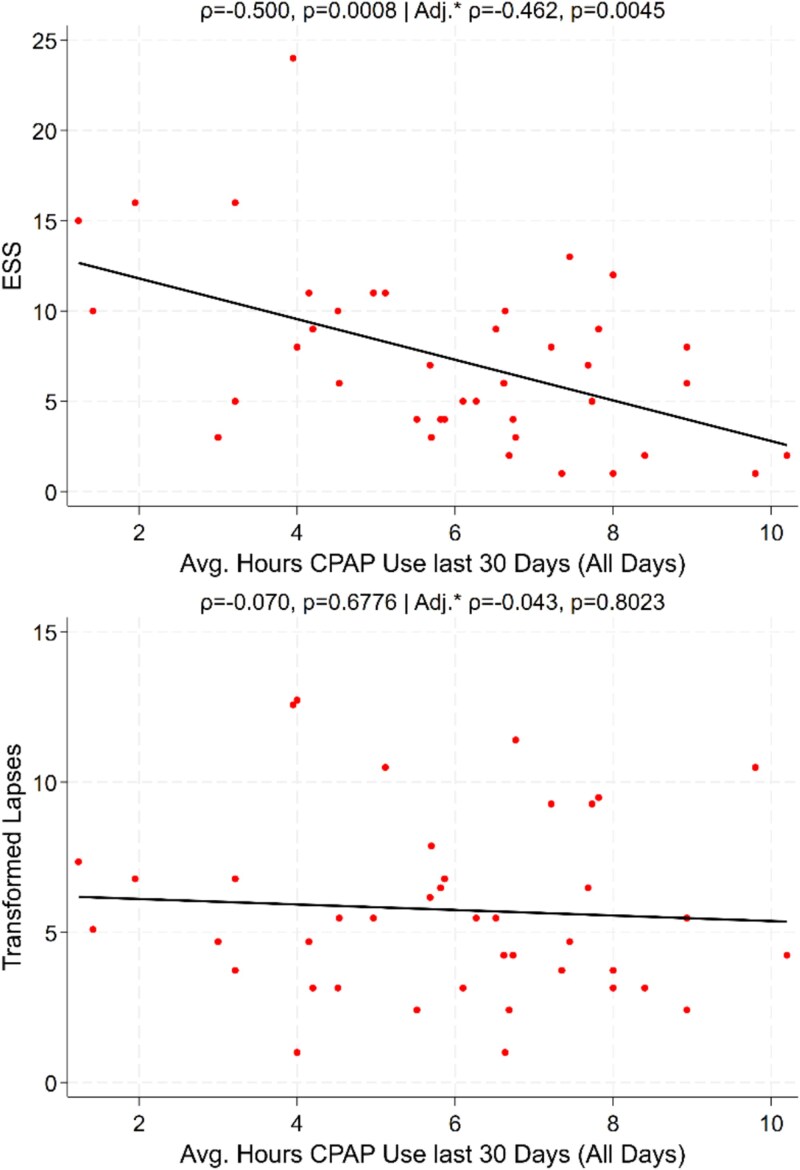
Associations between 30-day CPAP usage and sleepiness or attention/vigilance measures among participants with OSA using CPAP. In correlation analyses, we observed a significant association between greater CPAP usage and lower ESS scores, but no association between CPAP usage and PVT measures (see also Table 5). ^*^Adjusted for age, sex, and BMI.

Among the 41 patients with OSA and data on the amount of CPAP usage, greater hours/night of CPAP usage over the 30 days (adjusted rho = −0.46, *p* = .005) and 7 days (adjusted rho = −0.42, *p* = .011) were associated with lower ESS scores (Table 5; Figure 4). No correlations were found between the amount of CPAP use and measures from PVT.

**Table 5:** Correlations between CPAP usage and both ESS and PVT measurements

Measure	30-day average				7-day average				Previous day			
	*Unadjusted*		Adjusted^*^		*Unadjusted*		Adjusted^*^		*Unadjusted*		Adjusted^*^	
	*rho*	*P*	*rho*	*P*	*rho*	*P*	*rho*	*P*	*rho*	*P*	*rho*	*P*
ESS	**−0.50**	**.001**	**−0.46**	**.005**	**−0.45**	**.003**	**−0.42**	**.011**	−0.36	.019	−0.32	.057
Transformed lapses	−0.07	.678	−0.04	.802	−0.10	.535	−0.06	.735	−0.11	.498	−0.11	.524
Mean RRT	0.07	.664	0.07	.694	0.10	.553	0.08	.656	0.06	.702	0.07	.667

Statistically significant associations after Hochberg correction for thee sleepiness measures shown in **bold**. Abbreviations: AHI = Apnea Hypopnea Index; ODI = Oxygen Desaturation Index; PVT = Psychomotor Vigilance Test; ESS = Epworth Sleepiness Scale; RRT = Reciprocal Response Time.

^*^Partial correlation adjusted for age, sex, and BMI.

### Effects of CPAP Efficacy and Leak on Sleepiness

We also evaluated associations of residual AHI from the CPAP machines and device-specific leak metrics with sleepiness measures (see Table 6). Neither increased residual AHI nor worse leak metrics were associated with increased sleepiness on the ESS or worse performance on PVT. There was a significant, but unexpected, association between higher residual AHI and *better* performance on the PVT in our data; as only four patients had a residual AHI over the clinically used threshold of 10 events/hour [33], this result is both unanticipated and likely not clinically meaningful.

**Table 6:** Correlations between CPAP efficacy and both ESS and PVT measurements

Measure	**Residual AHI** ^**†**^ ^,^ ^**⁋**^				**Time in large leak** ^**‡**^ ^,^ ^**⁋**^				**95th percentile leak** ^**§**^ ^,^ ^**⁋**^			
	*Unadjusted*		Adjusted^*^		*Unadjusted*		Adjusted^*^		*Unadjusted*		Adjusted^*^	
	*rho*	*P*	*rho*	*P*	*rho*	*P*	*rho*	*P*	*rho*	*P*	*rho*	*P*
ESS	−0.13	.457	−0.22	.233	0.49	.127	−0.09	.851	0.14	.513	0.14	.542
Transformed lapses	−0.32	.057	**−0.50**	**.003**	0.04	.916	0.10	.838	0.12	.580	−0.13	.587
Mean RRT	0.37	.024	**0.50**	**.004**	0.17	.610	0.02	.974	−0.24	.255	−0.06	.791

Statistically significant associations after Hochberg correction for three sleepiness measures shown in **bold**. Abbreviations: AHI = Apnea–Hypopnea Index; ODI = Oxygen Desaturation Index; PVT = Psychomotor Vigilance Test; ESS = Epworth Sleepiness Scale; RRT = Reciprocal Response Time.

^*^Partial correlation adjusted for age, sex, and BMI;

^†^Residual AHI from CPAP machine (*n* = 37);

^‡^Minutes in large leak available from Philips devices (*n* = 11);

^§^95th percentile leak (L/min) available from ResMed devices (*n* = 24);

^⁋^Natural log-transformed for analysis.

### Effects of Sleep Duration on Sleepiness, OSA Severity, and CPAP Therapy

The mean self-reported sleep duration among all participants was 6.99 ± 1.65 hours (Table 1). Sleep duration was not associated with ESS scores or measures of attention/vigilance from PVT (Table S3). Sleep duration also did not correlate with OSA severity or PAP efficacy (Table S3), but was positively correlated with PAP usage, as assessed by both 30-day average use (adjusted rho = 0.49, *p* = .017) and 7-day average use (adjusted rho = 0.50, *p* = .014). Consistent with prior analyses, after adjustment for sleep duration, greater OSA severity and more PAP usage correlated with ESS but not PVT measures (Table S4).

## Discussion

This study reports on the relationships between subjective sleepiness based on the commonly used Epworth Sleepiness Scale and objective attention/vigilance as measured by the Psychomotor Vigilance Test in a sleep clinical population, as well as how each measure relates to disease severity and treatment among patients diagnosed with OSA. First, we found no meaningful associations between the ESS and PVT performance in a clinical population, suggesting that these two related measures capture distinct constructs among patients presenting to sleep clinics. The ESS assesses propensity to fall asleep, while the PVT measures sustained attention under monotonous conditions [40–42]. This important difference between sleepiness and attention/vigilance may explain the divergence in our findings. Second, we observed that worse sleepiness on the ESS was associated with more severe OSA and that those with greater CPAP usage had less sleepiness based on the ESS. In contrast, worse PVT performance was not significantly associated with greater OSA severity or less CPAP usage. These data have important implications with regard to evaluating “sleepiness” or “attention” among patients who are seen in sleep clinics.

The current results partially align with a prior study from Batool et al. [31] in which 100 participants referred to a sleep clinic for possible sleep apnea. Daytime sleepiness was evaluated using the ESS, and vigilance was measured with a portable 10-minute PVT device. The authors found that a poor PVT performance correlated with a higher ESS score, consistent with previously published literature [30, 32], and that OSA severity measured by AHI had no correlation with PVT measures. While our study found no meaningful associations between the PVT and ESS, in contrast to this prior study, we did find that OSA severity measured by AHI was correlated with ESS scores but not with PVT performance [43, 44]. Possible reasons for the differences in observed relationships between ESS and PVT in our study could be related to the length of PVT used, differences in PVT devices, methods of sleep evaluation, and different patient criteria.

Ultimately, our study provides interesting new data on the relationships between subjective sleepiness and objective attention/vigilance, with results suggesting that self-report of excessive sleepiness on ESS does not necessarily imply poor performance on PVT. Overall, our results confirm previous studies [24, 30, 45–47] suggesting that subjective and objective measures in patients with OSA should be assessed separately. These investigations [24, 31, 43] have similarly found poor correlations between the PVT and subjective sleepiness, leading them to conclude that objective and subjective measurements are not equivalent and should be used in conjunction. Although the ESS was not designed for item-level interpretation in clinical or research settings, whether individual items are more associated with objective alertness/vigilance may be an interesting avenue for future investigation.

Sleep duration represents an additional factor that may influence both subjective sleepiness and objective vigilance. Short sleep duration is established as a contributor to impaired attention [48, 49] and reaction time [50] and may also affect self-reported daytime sleepiness. In our sample, sleep duration was based on patient self-report at the time of the clinical visit and was available for a subset of participants. Adjustment for sleep duration did not meaningfully change the observed relationships between OSA severity, CPAP metrics, and measures of subjective or objective sleepiness. Importantly, we relied on self-reported sleep duration available in the EHR for these analyses, but future studies should assess whether more robust measurements of sleep duration (e.g. via sleep diaries or actigraphy) affect these observed associations.

While our comparison of the ESS and PVT suggests these measures may capture different constructs, our data also suggest that the ESS tracks more closely to OSA severity and treatment metrics than the PVT. We hypothesized that the PVT (since it is an objective measure) would be a better predictor of OSA severity and CPAP usage than the ESS; the observed data are contrary to this initial hypothesis. Patients with greater subjective sleepiness generally had more severe OSA, but there was no meaningful relationship between OSA severity and attention/vigilance as measured by the PVT. Consistent with these observations, within a subset of patients with OSA in whom data were available, we found that more CPAP usage was associated with less sleepiness based on the ESS but not better performance on the PVT. [51] Additional analyses showed that neither higher residual AHI nor greater leak (based on device-specific metrics) was associated with lower ESS or worse PVT performance. Therefore, making clinical recommendations based on a high residual AHI or a large mask leak, at least in terms of EDS, may not be clinically warranted.

The ESS is commonly used in sleep practices to measure sleepiness, and our data support continuing to use the ESS in evaluating patients with OSA for daytime sleepiness. On the other hand, the lack of association between PVT and OSA severity/treatment does not strongly support a recommendation for routine use of the 3-minute PVT in patients being evaluated clinically for OSA. The 3-minute PVT was selected due to the time constraints of clinical encounters and our prior work demonstrating an acceptable tradeoff between effect size and testing duration: while the 10-minute PVT showed a 22.7% greater average effect size, the 3-minute version reduced testing time by 70%, which we considered an appropriate balance [27]. However, the duration of the 3-minute PVT may be insufficient to capture “sleepiness” reported by patients in a clinical setting. It is possible that the 5- or 10-minute versions of the PVT [52, 53] may provide more accurate measures of sustained attention and vigilance than the 3-minute version used in our study. Evidence on the relative sensitivity of the 3-minute versus 10-minute PVT is mixed—some studies suggest that the shorter version is equally sensitive [54], while others have found the 3-minute PVT did not produce results comparable to the 5- or 10-minute PVT in elite athletes, arguing that the two are not interchangeable [55]. Additionally, in individuals with mild OSA, the 3-minute PVT may lack the sensitivity needed to detect subtle deficits in vigilance or sleepiness. Future research could consider comparing PVTs of longer duration to assess whether consistent results are obtained.

Another consideration is that our study examined sleepiness in a clinical population at only one point in time. Performing multiple PVT assessments over a short period (e.g. several days or even multiple times in the same day) may better characterize a patient’s attention/vigilance. Moreover, longitudinal studies over weeks to months evaluating sleepiness using the ESS and/or PVT at multiple time points would likely better characterize the response to treatments of various sleep disorders. The lack of learning effect with the PVT [56] provides an advantage over potential bias in self-reported measures of sleepiness with longitudinal evaluations. The technology to remotely assess PVT exists [57, 58] and is rapidly developing, although more work is needed to establish the reliability of these applications.

Having an objective longitudinal biomarker that reflects patient-reported symptoms could better inform treatment decisions, particularly in patients in whom the self-reported symptoms are less reliable (e.g. commercial drivers [59–61] or patients with unrecognized symptoms [19]). Repeatedly measuring such a biomarker over time could also change how we determine outcome benefits when treatments are initiated or changed in patients treated for various sleep disorders. For instance, treatment for patients with narcolepsy is initiated primarily based on costs and side effects, not efficacy, since there are no head-to-head trials of the commonly used narcolepsy medications. Demonstrating objective improvements in sleepiness may facilitate an outcomes-based approach to support specific treatment options in narcolepsy and other sleep disorders. For example, does CBT-I or hypnotics in participants with insomnia or treating periodic limb movements or restless leg syndrome (RLS) improve the PVT or ESS over time? Future studies in larger samples with repeated outcome measures in participants with a variety of sleep disorders should answer these questions.

This study has several limitations. Most participants had never previously completed a PVT. While the task is not known to be affected by practice effects, performance may still vary due to initial unfamiliarity. A 30-second practice trial was offered before the actual test, which most participants performed. Additionally, the majority of participants were being evaluated or treated for OSA, but the sample also included patients with insomnia, parasomnias, circadian rhythm disorders, and hypersomnia (narcolepsy and idiopathic hypersomnia). The AHIs were derived from HSATs and PSGs, and used interchangeably. We found no significant differences in our primary findings after stratifying our analyses by type of sleep study. The majority of sleep studies scored hypopneas using the 4% oxygen desaturation criterion. Prior work has shown that patients classified as having OSA by the 3% desaturation or arousal rule exhibit slightly higher ESS scores than those without OSA [62], although this mean difference was less than one point. Nonetheless, repeating our analyses in cohorts scored using the 3% desaturation criterion warrants further investigation.

Future studies should include a larger population of patients with a variety of clinical sleep disorders followed longitudinally to better capture the association between subjective sleepiness and objective attention/vigilance, including within different patient subgroups or for different conditions. While the study provides new information on the relationships between these measures within patients from an academic sleep practice, not all patients seen in the timeframe were approached or completed the PVT. This could reduce generalizability, depending on how the characteristics of included participants compare to those who did not participate. Relatedly, the sample was enriched for patients with diagnosed OSA, and results may be less generalizable to other sleep disorders. CPAP usage was not associated with PVT outcomes; however, the absence of pre- and post-CPAP comparisons limits our ability to infer a causal relationship between therapy and outcomes. As with any prospective study, there may also be unmeasured covariates that influence the relationship between patient-reported sleepiness and/or objective performance, which were not readily available in our sample. Finally, we had a relatively small sample size for certain analyses; at an α of 0.05, our sample had 80% power to detect correlations of >0.21 when comparing ESS and PVT among all participants and >0.42 when associating CPAP usage with sleepiness measures. Thus, non-significant results for smaller correlations should be interpreted with some caution and, more generally, all results interpreted with respect to the clinical significance of associations (e.g. rho of 0.1, 0.3, and 0.5 representing small, medium, and large effects [37]). Ultimately, future studies with larger sample sizes would be beneficial to both validate associations found here and to understand how sample heterogeneity influences results. Additionally, psychometric analysis can be done with activity-specific ESS items to see which may correlate better with PVT outcomes rather than the total ESS score.

There are also important strengths that should be emphasized. The present study provides insights into the relationships between subjective sleepiness and objective attention/vigilance among a representative (and heterogeneous) sample of participants seen at an academic clinical sleep center, enhancing generalizability. Other strengths include performing the PVT and ESS at the same visit in a diverse clinical sample, including patients with different sleep disorders (e.g. not only patients with sleep apnea), and the availability of objective data on CPAP usage and efficacy to explore associations between treatment and sleepiness in patients with OSA.

## Conclusions

Among patients evaluated in an academic clinical sleep center, we found no meaningful associations between subjective sleepiness as measured by the ESS and objective attention/vigilance as measured by the PVT. These data suggest that the PVT and ESS may be characterizing two distinct aspects of sleepiness/function experienced by patients. Among those diagnosed with OSA, our data indicate that the ESS correlates more closely with disease severity and treatment metrics than the PVT. Patients with more severe OSA demonstrated worse subjective sleepiness on the ESS, but not worse attention/vigilance based on the PVT. Similarly, greater CPAP usage was associated with better subjective sleepiness but not associated with better performance on the PVT. Additional studies are needed to determine whether the PVT has utility as an outcome in patients with OSA.

## Supplementary Material

zsag094_PVT_supplementary_file_final
