# Supplementary material for: Sleepiness and attention in sleep-clinic patients and their associations with apnea severity and treatment
Source: Sleep. 2026 May 15;49(7):zsag094. doi: 10.1093/sleep/zsag094 (PMC13357509; doi:10.1093/sleep/zsag094)
Supplement: zsag094_PVT_supplementary_file_final [file zsag094_pvt_supplementary_file_final.docx]

**Sleepiness and Attention in Sleep-Clinic Patients and their Associations with Apnea Severity and Treatment**

Allison Schwab^1^

Brendan T. Keenan^1^

Nathan C. Nowalk.^1^

Mathias Basner^2^

Charles Bae^1^

*^1^Division of Sleep Medicine/Department of Medicine, University of Pennsylvania Perelman School of Medicine, Philadelphia, PA, USA*

*^2^Unit for Experimental Psychiatry, Division of Sleep and Chronobiology, University of Pennsylvania Perelman School of Medicine, Philadelphia, PA, USA*

**Corresponding Author:**

Charles Bae, MD

Division of Sleep Medicine

University of Pennsylvania Perelman School of Medicine

3624 Market Street, Suite 205

Philadelphia, PA 19104

Email: charles.bae@pennmedicine.upenn.edu

**Supplemental Tables**

**Table S1**. Correlations between ESS and PVT measurements Excluding Treated OSA patients

| **PVT Measure** | **ESS of All Participants** | | | | **ESS of Diagnosed OSA** | | | |
| --- | --- | --- | --- | --- | --- | --- | --- | --- |
|  | ***Unadjusted*** | | ***Adjusted**** | | ***Unadjusted*** | | ***Adjusted**** | |
|  | *rho* | *p* | *rho* | *p* | *rho* | *p* | *rho* | *p* |
| Transformed Lapses | 0.08 | 0.368 | 0.08 | 0.384 | 0.09 | 0.431 | 0.11 | 0.373 |
| Mean RRT | -0.07 | 0.432 | -0.05 | 0.593 | -0.08 | 0.533 | -0.05 | 0.669 |
| *Partial correlation adjusted for age, sex and BMI; Abbreviations: PVT = Psychomotor Vigilance Test; ESS = Epworth Sleepiness Scale; RRT = Reciprocal Response Time | | | | | | | | |

**Table S2.** Correlations between OSA Severity and both ESS and PVT measurements based on type of sleep study performed

| **Measure** | **AHI** | | | | **SpO_2_ nadir** | | | | **Minutes SpO_2_ < 90%^†^** | | | |
| --- | --- | --- | --- | --- | --- | --- | --- | --- | --- | --- | --- | --- |
|  | ***Unadjusted*** | | ***Adjusted**** | | ***Unadjusted*** | | ***Adjusted**** | | ***Unadjusted*** | | ***Adjusted**** | |
|  | *rho* | *p* | *rho* | *p* | *rho* | *p* | *rho* | *p* | *rho* | *p* | *rho* | *p* |
| **PSG (n = 46)** | | | | | | | | | | | | |
| ESS | 0.35 | 0.017 | 0.20 | 0.203 | -0.30 | 0.046 | -0.12 | 0.448 | **0.40** | **0.006** | 0.30 | 0.057 |
| Transformed Lapses | 0.14 | 0.357 | 0.09 | 0.589 | -0.30 | 0.046 | -0.23 | 0.135 | 0.26 | 0.080 | 0.21 | 0.190 |
| Mean RRT | -0.15 | 0.329 | 0.01 | 0.971 | 0.25 | 0.099 | 0.12 | 0.467 | -0.16 | 0.297 | -0.04 | 0.827 |
| **HSAT (n = 25)** | | | | | | | | | | | | |
| ESS | 0.46 | 0.022 | 0.47 | 0.032 | -0.46 | 0.026 | -0.45 | 0.051 | 0.40 | 0.051 | 0.44 | 0.052 |
| Transformed Lapses | -0.27 | 0.190 | 0.02 | 0.934 | -0.01 | 0.980 | 0.16 | 0.503 | -0.05 | 0.823 | 0.09 | 0.696 |
| Mean RRT | 0.25 | 0.234 | -0.06 | 0.800 | 0.00 | 0.986 | -0.08 | 0.750 | 0.01 | 0.960 | -0.20 | 0.409 |
| Statistically significant associations after Hochberg correction for 3 sleepiness measures shown in **bold**. *Partial correlation adjusted for age, sex and BMI; †natural log transformed for analyses; Abbreviations: ESS = Epworth Sleepiness Scale; RRT = Reciprocal Response Time; AHI = apnea-hypopnea index; SpO2 = oxygen saturation; PSG = polysomnography; HSAT = home sleep apnea test | | | | | | | | | | | | |

**Table S3**. Correlations between self-reported sleep duration and study measures

| **Study Measure** | **Sleep Duration** | | | | |
| --- | --- | --- | --- | --- | --- |
|  | ***N*** | ***Unadjusted*** | | ***Adjusted**** | |
|  |  | *rho* | *p* | *rho* | *p* |
| *Sleepiness/Attention* |  |  |  |  |  |
| ESS | 134 | -0.10 | 0.236 | -0.10 | 0.281 |
| Transformed Lapses | 134 | -0.08 | 0.333 | -0.08 | 0.380 |
| Mean RRT | 134 | 0.08 | 0.380 | 0.07 | 0.452 |
| *OSA Severity* |  |  |  |  |  |
| AHI^†^ | 60 | -0.04 | 0.739 | 0.09 | 0.489 |
| SpO_2_ nadir^†^ | 57 | 0.25 | 0.064 | 0.16 | 0.246 |
| Minutes SpO2 < 90%**^†,§^** | 58 | -0.22 | 0.102 | -0.08 | 0.579 |
| *PAP Usage* |  |  |  |  |  |
| 30-day Average**^‡^** | 28 | 0.42 | 0.027 | **0.49** | **0.017** |
| 7-day Average**^‡^** | 28 | 0.40 | 0.036 | **0.50** | **0.014** |
| Previous Day**^‡^** | 28 | 0.31 | 0.110 | 0.37 | 0.081 |
| *PAP Efficacy* |  |  |  |  |  |
| Residual AHI**^‡,§^** | 25 | -0.01 | 0.964 | -0.21 | 0.376 |
| Time in Large Leak**^‡,§^** | 9 | 0.08 | 0.846 | -0.04 | 0.947 |
| 95^th^ Percentile Leak**^‡,§^** | 14 | 0.09 | 0.761 | -0.19 | 0.581 |
| Statistically significant associations after Hochberg correction within each variable domain shown in **bold**. *Partial correlation adjusted for age, sex and BMI; †Analyses performed in patients with OSA not on treatment at time of PVT/ESS; ‡Analyses performed on those with OSA on PAP; **^§^**natural log transformed for analyses; Abbreviations: ESS = Epworth Sleepiness Scale; RRT = Reciprocal Response Time; AHI = apnea-hypopnea index; SpO_2_ = oxygen saturation. | | | | | |

**Table S4.** Associations of OSA severity and PAP usage and efficacy measures with ESS and PVT after adjustment for sleep duration

| **Measure** | **N** | **ESS** | | | | **Transformed Lapses** | | | | **Mean RRT** | | | |
| --- | --- | --- | --- | --- | --- | --- | --- | --- | --- | --- | --- | --- | --- |
|  |  | ***Unadjusted**** | | ***Adjusted*** ^†^ | | ***Unadjusted**** | | ***Adjusted*** ^†^ | | ***Unadjusted**** | | ***Adjusted*** ^†^ | |
|  |  | *rho* | *p* | *rho* | *p* | *rho* | *p* | *rho* | *p* | *rho* | *p* | *rho* | *p* |
| *OSA Severity* |  |  |  |  |  |  |  |  |  |  |  |  |  |
| AHI | 60 | **0.42** | **0.001** | 0.29 | 0.030 | 0.07 | 0.597 | 0.12 | 0.377 | -0.11 | 0.422 | -0.14 | 0.300 |
| SpO_2_ nadir | 57 | **-0.32** | **0.016** | -0.10 | 0.475 | -0.23 | 0.091 | -0.26 | 0.064 | 0.25 | 0.062 | 0.28 | 0.041 |
| Minutes SpO2 < 90%^‡^ | 58 | **0.35** | **0.008** | 0.25 | 0.072 | 0.21 | 0.112 | 0.17 | 0.214 | -0.22 | 0.097 | -0.16 | 0.249 |
| *PAP Usage* |  |  |  |  |  |  |  |  |  |  |  |  |  |
| 30-day Average | 28 | **-0.61** | **0.001** | **-0.70** | **0.0003** | -0.05 | 0.797 | -0.18 | 0.434 | 0.03 | 0.881 | 0.19 | 0.401 |
| 7-day Average | 28 | **-0.46** | **0.014** | **-0.52** | **0.012** | -0.07 | 0.712 | -0.19 | 0.406 | 0.02 | 0.921 | 0.16 | 0.478 |
| Previous Day | 28 | -0.38 | 0.048 | -0.33 | 0.128 | -0.22 | 0.255 | -0.34 | 0.122 | 0.10 | 0.625 | 0.25 | 0.268 |
| *PAP Efficacy* |  |  |  |  |  |  |  |  |  |  |  |  |  |
| Residual AHI^‡^ | 25 | -0.17 | 0.404 | -0.36 | 0.125 | -0.23 | 0.277 | -0.33 | 0.174 | 0.33 | 0.106 | 0.39 | 0.104 |
| Time in Large Leak^‡^ | 9 | 0.50 | 0.172 | -0.28 | 0.725 | -0.14 | 0.718 | 0.09 | 0.908 | 0.35 | 0.362 | 0.09 | 0.908 |
| 95^th^ Percentile Leak^‡^ | 14 | -0.25 | 0.394 | -0.21 | 0.570 | 0.15 | 0.606 | 0.06 | 0.876 | -0.36 | 0.209 | -0.19 | 0.600 |
| Statistically significant associations after Hochberg correction for 3 sleepiness measures shown in **bold**. *Unadjusted correlation restricted to the subset of patients with non-missing sleep duration; ^†^Partial correlation adjusted for age, sex, BMI and sleep duration; ^‡^natural log transformed for analyses; Abbreviations: PVT = Psychomotor Vigilance Test; ESS = Epworth Sleepiness Scale; RRT = Reciprocal Response Time | | | | | | | | | | | | | |
